# Subject-specific timing adaption in time-encoded arterial spin labeling imaging

**DOI:** 10.1007/s10334-023-01121-y

**Published:** 2023-09-28

**Authors:** Nora-Josefin Breutigam, Daniel Christopher Hoinkiss, Simon Konstandin, Mareike Alicja Buck, Amnah Mahroo, Klaus Eickel, Federico von Samson-Himmelstjerna, Matthias Günther

**Affiliations:** 1 Imaging Physics, Fraunhofer Institute for Digital Medicine MEVIS, Max-von-Laue-Str. 2, 28359 Bremen, Germany; 2 Mediri GmbH, Heidelberg, Germany; 3 Faculty 1 (Physics/Electrical Engineering), University of Bremen, Bremen, Germany; 4 Bremerhaven University of Applied Science, Bremerhaven, Germany

**Keywords:** Arterial spin labeling, Arterial transit delay artifacts, Free-lunch approach, Time-encoded pCASL, Subject-specific timing

## Abstract

**Objectives:**

One challenge in arterial spin labeling (ASL) is the high variability of arterial transit times (ATT), which causes associated arterial transit delay (ATD) artifacts. In patients with pathological changes, these artifacts occur when post-labeling delay (PLD) and bolus durations are not optimally matched to the subject, resulting in difficult quantification of cerebral blood flow (CBF) and ATT. This is also true for the free lunch approach in Hadamard-encoded pseudocontinuous ASL (H-pCASL).

**Material and methods:**

Five healthy volunteers were scanned with a 3 T MR-system. pCASL-subbolus timing was adjusted individually by the developed adaptive Walsh-ordered pCASL sequence and an automatic feedback algorithm. The quantification results for CBF and ATT and the respective standard deviations were compared with results obtained using recommended timings and intentionally suboptimal timings.

**Results:**

The algorithm individually adjusted the pCASL-subbolus PLD for each subject within the range of recommended timing for healthy subjects, with a mean intra-subject adjustment deviation of 47.15 ms for single-shot and 44.5 ms for segmented acquisition in three repetitions.

**Discussion:**

A first positive assessment of the results was performed on healthy volunteers. The extent to which the results can be transferred to patients and are of benefit must be investigated in follow-up studies.

**Supplementary Information:**

The online version contains supplementary material available at 10.1007/s10334-023-01121-y.

## Introduction

Perfusion is an important parameter for the diagnosis of neurological diseases, which are often accompanied or preceded by changes in blood flow. Arterial spin labeling (ASL) [1] is a noninvasive method for measuring organ perfusion using magnetic resonance imaging (MRI). It uses the arterial blood itself as an endogenous tracer. To this end, ASL uses radio frequency (RF) pulses to magnetically “label” the arterial blood before entering an organ. Thereby allowing the measurement of various hemodynamic parameters such as cerebral blood flow (CBF) (perfusion), arrival times such as arterial transit times (ATT) and arterial blood volume, as well as certain exchange parameters such as vessel wall permeability [2, 3].

There are several ASL techniques [1, 4–6], with pseudocontinuous ASL (pCASL) [7–9] now established as the quasi-standard for steady-state perfusion imaging with ASL [10]. Here, a bolus is created over a period of time (typically 1–3 s) by magnetically labeling blood as it flows through the labling plane outside the imaging volume by locally inverting the magnetization. During a certain delay time, the labeled blood flows downstream and migrates to the tissue [1].

An important physiological parameter for ASL measurements of brain perfusion is ATT. This is the time required for blood to travel from the labeling plane to the imaging voxels of the tissue [11, 12]. ATT is an individual local parameter [13] and therefore important for accurate quantification of absolute CBF [11, 14–16]. If the ATTs are prolonged, artifacts called arterial transit delay (ATD) artifacts may occur [12], complicating correct clinical diagnosis, e.g., in old patients, in patients with ischemic stroke, steno-occlusive disease, or moyamoya disease [11, 12, 17–19]. ATD artifacts are promoted by non-optimal timing of bolus durations (BD) and post-labeling delays (PLD). This is due to the fact that with prolonged ATT, the defined PLD for the image acquisition is too short. The perfusion image then shows a high signal of labeled blood still in the macrovascular phase that has not yet reached the capillary exchange site. Although there are recommended timings for certain age groups and diseases [15], it is not possible to determine the extent to which individual ATTs deviate from these before the start of the acquisition of the perfusion scan. Clinically, information on ATT can be used to detect hemodynamic disturbances in stroke, vascular occlusion or malformations, and aging processes [20]. Several approaches exist to determine ATTs.

A common method for determining ATT is to sample the ASL signal over time and fit a parametric model curve to this sampled data [14]. Such fits yield parameter maps for ATT which can be used to correct CBF values for prolonged ATTs. In classical multi-TI/PLD ASL, multiple images are acquired in multiple measurements each using a different inflow time (TI) or PLD to temporally sample the ASL signal. However, compared to single-TI/PLD-ASL, this approach significantly increases the acquisition time (TA) [21]. Repetitions of the multi-TI/PLD measurement to achieve a sufficiently high signal-to-noise ratio (SNR) further increases the TA. The time constraints of a clinical setup often limit the applicable number of TIs/PLDs, which in turn may reduce the accuracy of quantified perfusion parameters.

Hadamard (or time-encoded) pCASL (H-pCASL) was proposed as a time and temporal SNR efficient multi-TI/PLD technique to accurately sample the ASL signal [22–24]. However, a potential disadvantage of H-pCASL in general is that all N-encoded images are required to decode each perfusion-weighted image (PWI). Artifacts in one or more of the encoded images carry over to all decoded images, potentially leading to erroneous results [21, 25–27]. Furthermore, with standard H-pCASL all N-encoded images have to be acquired before the decoding process of PWIs can be performed. Walsh-Ordered H-pCASL (WH-pCASL) [28] tries to solve this using a specific ordering of the encoding steps such that intermediate PWIs can be decoded even when only early subsets of all N-encoded images are available.

To optimize the timing of the ASL measurement and to obtain ATT information without time penalty and with theoretically the same SNR in CBF maps as in standard pCASL measurements, the so-called “free lunch” (FL) approach for H-pCASL/ WH-pCASL was presented [21]. Here one selects a longer duration and PLD for the first (sub)bolus (further called pCASL-subbolus) than for subsequent subboli (typically, similar to a standard pCASL scan). During the long PLD, the remaining subboli can be generated. This way, not changing the total scan time, a conventional ASL perfusion image resulting from the first bolus and, as a “free lunch”, further perfusion images for determining ATT from the remaining subboli and their respective PLDs are obtained.

The approaches described can reduce but not completely eliminate the aforementioned ATD artifacts without extending the times for TI/PLD sufficiently to account for delayed ATTs as a precaution. This would lead to a significant increase in measurement time and would be at the expense of SNR. In the FL approach, for example, the longer pCASL-subbolus image ideally shows a fully perfused brain, while the remaining shorter subboli are used to sample the different inflow phases of the labeled blood on its way to the capillary exchange site. The recommended timings [15, 21] for healthy subjects are adequate in most cases. However, when ATT is prolonged, the pCASL-subbolus image exhibits ATD artifacts. Strategies such as increasing the maximum TI and/or decreasing the pCASL-BD work only to a limited extent because the signal decreases with T_1_ relaxation, and shortening the pCASL-subbolus degrades the SNR. Therefore, it is desirable to have a pCASL-BD as long as possible, but short enough to avoid ATD artifacts and leave enough time for the remaining subboli. Consequently, it may be advantageous to use individualized timing for each patient to achieve optimal sampling and accurate quantification of CBF.

Early detection of non-optimal timing of BD and PLD is possible with the proposed WH-pCASL, where the first few images already yield usable information. However, PLD, net BD, and individual subbolus durations (SBD) are protocol parameters fixed at sequence start. To obtain optimal results, either a low-resolution pre-scan is required [29] or at least one of these measurement parameters must be changed during runtime. We propose an acquisition strategy allowing automatic adjustment of SBDs during the measurement. This can be performed in a very efficient way.

Preliminary development steps of this work have been presented in abstract form [30–32].

## Theory

This section explains the theoretical background of how automatic adjustment of timing in a time-encoded pCASL acquisition is possible without significantly increasing the measurement time or unnecessarily shortening the pCASL-subbolus.

**Fig. 1:**
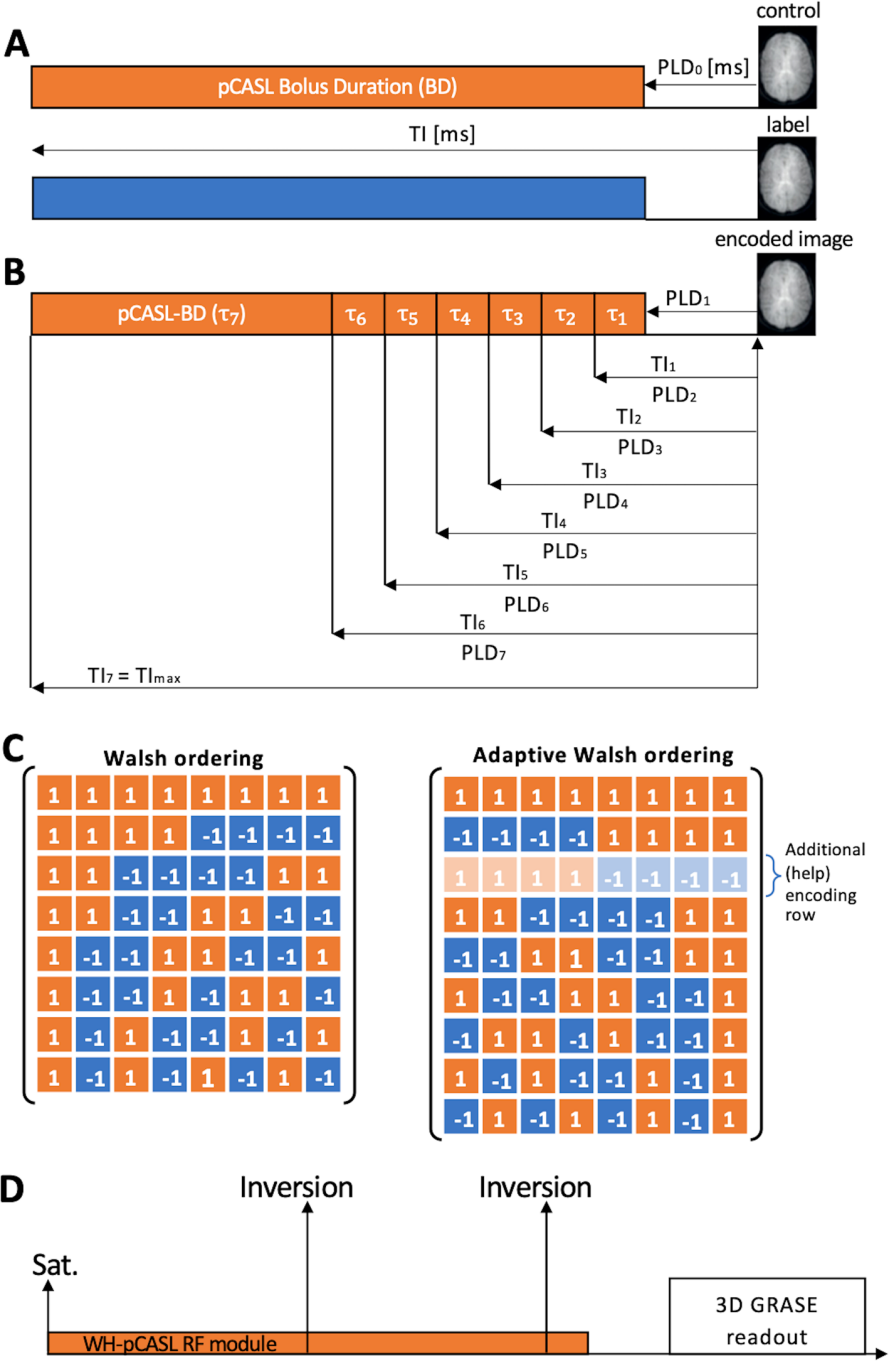
**A** Standard pCASL acquisition with long control (orange) and label (blue) bolus. Start PLD_0_ and pCASL-bolus duration (BD) define the inflow time (TI). **B** Splitting of the longer bolus in multiple subboli in a time-encoded pCASL experiment. Each subbolus has a different duration ${\tau }_{i}$ (*i* = 1–7), post labeling delay (PLD_*i*_) and inflow time (TI_*i*_). Again, respective PLD_i_ and duration ${\tau }_{i}$ determine TI_*i*_ and vice versa. **C** The conventional Walsh ordering of an 8 × 8 Hadamard matrix is converted to the “adaptive” version to facilitate subbolus duration (SBD) adjustments based of intermediate perfusion-weighted images. In addition, the second encoding row is used twice, but with the control (“1”) and labeling (“−1”) phases reversed. This row is skipped in the final decoding process in which “1” stands for addition and “−1” for subtraction. **D** General sequence scheme with one saturation and two background suppression pulses

In Hadamard-encoded pCASL imaging, the long pCASL-bolus (Fig. 1A) is separated into several so-called subboli (Fig. 1B). Each subbolus can have a different duration ${\uptau }_{i}$. Consequently, each subbolus has a different TI_*i*_ and PLD_*i*_ (*i* = 1–7). In the FL-approach the subbolus with the longest inflow time (TI_max_) still represents the long pCASL-(sub)bolus and the remaining subboli are used to sample the inflow of the blood. Accordingly, the blood flowing into the brain is marked with a pattern of label and control states corresponding to the entries of a Hadamard matrix (Fig. 1C). Each entry of “1” or “−1” stands for a subbolus with the status “control” or “label”. The perfusion-weighted images (PWI)s with the different TIs can be decoded by adding (1) and subtracting (−1) the encoded images according to the entries of the Hadamard columns.

**Fig. 2:**
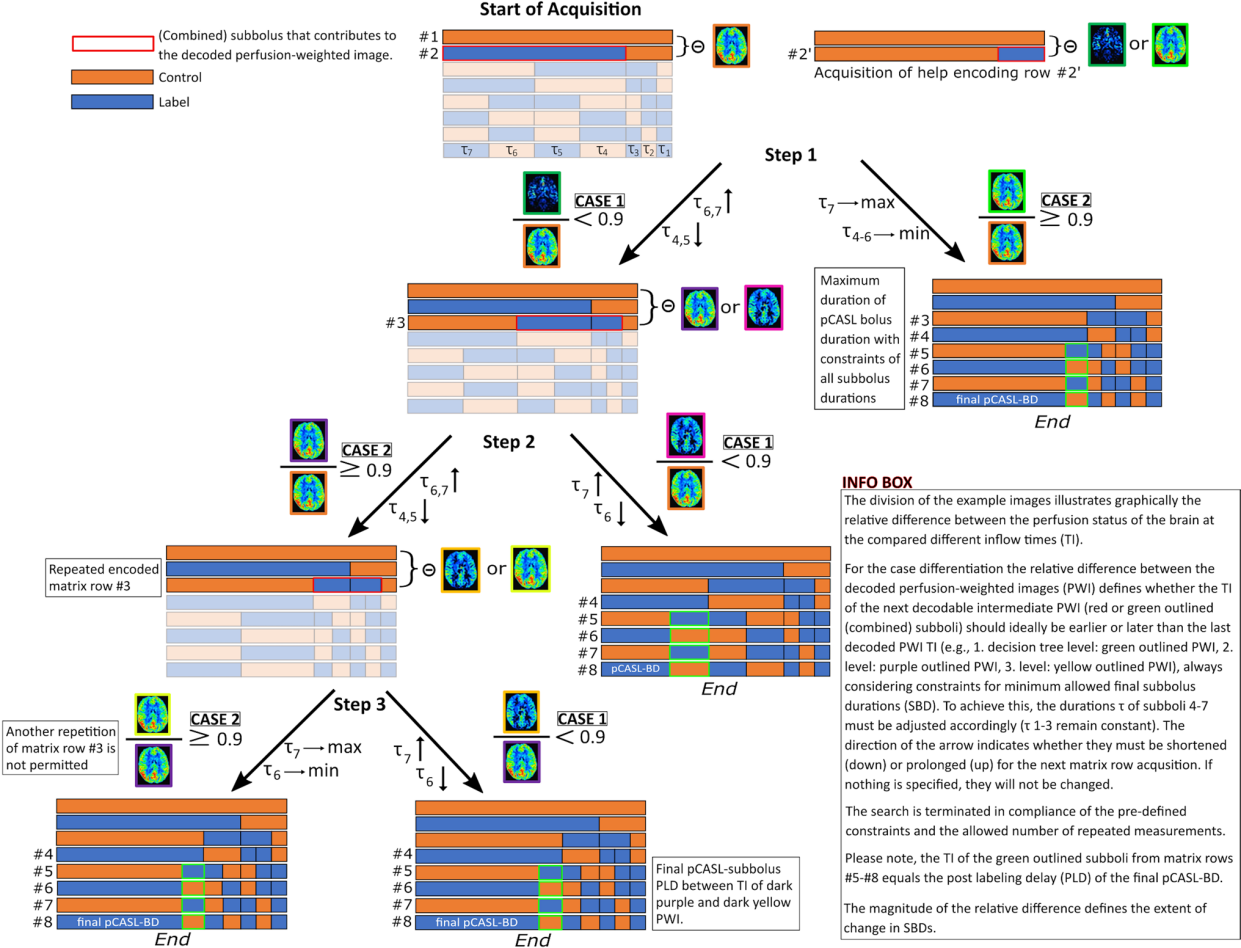
The decision tree shows representatively the algorithm for stepwise approximation of an individual final pCASL subbolus duration (SBD) in a time-encoded pCASL measurement using the adapted 8 × 8 Hadamard encoding matrix (only 7 SBDs shown here because the 8th column does not result in a perfusion-weighted image (PWI)). Crucial to the algorithm is the fact that neighboring subboli with the same label or control state can be combined into longer subboli. Within these combined subboli, the subsequent borders for a change of label and control are not yet fixed. This results in initial matrices with smaller dimensions, which can be used for the calculation of here so-called intermediate PWIs. After the initial 2 × 2 acuqisition (#1 and #2) and using the help encoding row (#2′) PWIs with the longest inflow time (TI) (orange PWI) and with an earlier TI (green PWIs) can be calculated. Depending on the subject measured, the earlier PWIs may show a brain with more (dark green PWI) or less (light green PWI) labeled blood inside. Then, the ratio of the number of voxels above the noise threshold is determined and compared in a complete image volume of PWIs (only one slice is shown here as an example). Depending on the ratio, the SBDs of the next Hadamard matrix encoding row(s) are adjusted (see Info Box). The exact calculations of the SBDs can be found in Online Resource 2. Depending on the result of the PWI comparison and the previously defined allowed maximum and minimum duration of each subboli, individual rows are adjusted, repeated or the measurement is terminated with adjusted SBDs by including all subsequent matrix rows accordingly

When Walsh-ordering a natural-ordered Hadamard matrix, the individual rows of the matrix are ordered by their sequence, i.e. the number of sign changes in their entries. Adjacent subboli being in the same phase (label or control) effectively form one combined longer subbolus. Consequently, the first two rows of any Walsh-ordered Hadamard matrix correspond to a 2 × 2 natural-ordered one, the first four rows correspond to a 4 × 4 matrix, the first eight rows correspond to an 8 × 8 matrix, etc. (compare Fig. 2, Online Resource 1 Figure S2).

It is important to note that within these combined longer partial boluses each individual duration of the shorter subboli is not yet fixed. This degree of freedom is exploited in the adaptive WH-pCASL imaging method. By applying a dedicated encoding matrix (Fig. 1C right), the duration of the subboli, which are decisive for the final pCASL-BD, can be adjusted during active scanning.

### Adaptive bolus duration algorithm

The underlying algorithm is shown in Fig. 2. In each step two intermediate PWIs with different TIs are compared and analyzed during the measurement’s runtime to iteratively adapt the pCASL-BD/pCASL-PLD_7_ while keeping the number of acqusitions as low as possible. Depending on how much blood is supplied to the brain in each intermediate PWI, the SBDs of the subsequent matrix rows are either shortened or prolonged.

At the beginning of the algorithm TI_max_ and the first PLD_1_ are set (Fig. 2, Start of Acquisition). These parameters cannot be changed during the runtime of the measurement. Furthermore, the start SBDs are defined, whereby only the inflow time TI_3_ (first change between label and control state in #2 & #2′) is fixed at this stage (Figs. 1B, C, 2, acquisition of help encoding row). Moreover, the SBDs ${\uptau }_{1-3}$ remain constant throughout the duration of the acquisition.

After the acquisition of further encoding matrix rows (Fig. 2, Step 1–3), it can be seen that with each additional row of the encoding matrix, further (combined) subboli are given fixed durations that cannot be changed during the acquisition of subsequent matrix rows. Whenever there is a change between label and the control state, the corresponding TI is fixed for the course of the experiment. If this defined TI is to be changed, the acquisition of the corresponding row in the encoding matrix must be repeated. This results in the essential property of the presented algorithm. The duration of the final pCASL-subbolus (${\uptau }_{7}$) and thus the decoded PWI can not be investigated with the acquisitions of single encoding matrix rows. With the 8 × 8 encoding matrix used, the pCASL-subbolus PWI can only be decoded when all eight matrix rows have been successfully acquired. As a result, a stepwise adaptation to a final pCASL-PLD_7_ is performed during the experiment by comparing PWI with earlier TI with PWI with later TI and adjusting the individual SBDs in the subsequent matrix rows accordingly. Within few iteration steps, a more “optimized” SBD of the final pCASL-subbolus can thus be approximated.

The number of adjustments is limited by the size of the used encoding matrix, which is two for an 8 × 8 Hadamard acquisition matrix.

All decisions are based on the “degree of filling” of the brain. If the brain is still sparsely filled, it is assumed that labeled blood continues to be in the macrovascular phase. Therefore, for the comparison, the number of voxels having signal values above the noise level of the PWI is calculated. The histogram of each image slice is used to calculate the respective noise level. Here, the observation is exploited that the number of “filled” voxels (NoV) increases with TI (Online Resource Figure S1), at least until venous outflow and/or until SNR decreases too much. To compare the NoV in the complete image volume of two PWIs, the ratio between them is calculated, e.g., in Step 1 (Fig. 2):

$${R}_{\mathrm{rel},1}=\frac{\mathrm{NoV}\left({\mathrm{PWI}}_{\mathrm{TIearly}}\right)}{\mathrm{NoV}\left({\mathrm{PWI}}_{\mathrm{TImax}}\right)}.$$

A threshold value is used as a criterion for adjusting the SBDs to reduce potential ATD artifacts in the final pCASL-subbolus PWI. If *R*_rel_ < threshold, the potential final pCASL-BD has to be shortened. If *R*_rel_ ≥ threshold, the potential final pCASL-BD has to be prolonged, which means a repeated acquisition of the previous matrix row with adapted timings is necessary. *R*_rel_ is used for the calculation of the adapted SBDs as well.

In the following, the flow of the algorithm is explained step by step on the basis of Fig. 2.

**Step 1:**

Since only one PWI can be calculated with the first two encoded images, an auxiliary row with an interchanged label and control state of the second matrix row is included accordingly (#2′). This row is skipped in the final decoding process. Two PWIs, one with TI_max_ (PWI#1, orange) and one with an earlier TI (PWI#2, green) can then be compared.

If ${R}_{\mathrm{rel},1}\ge$ threshold (CASE 2), the brain is already perfused early. To increase the SNR of the final pCASL-subbolus, the final pCASL-BD should become maximally long (minimum PLD) and all other subboli being given a minimum duration within defined limits. All remaining matrix rows are acquired and no further adjustments are made. Any adjustment is made within a defined allowed minimum and maximum durations for the last pCASL-subbolus and the remaining subboli (see “Methods” section).

If ${\mathrm{R}}_{\mathrm{rel},1}<$ threshold (CASE 1), the brain is not as much perfused in PWI#2 than in PWI#1. To reduce potential ATD artifacts in the final pCASL-PWI, the third matrix row (#3) is acquired with adapted subboli durations. The relative difference in the number of filled voxels defines how long the individual (combined) subboli in this matrix row become:

$${\mathrm{SBD}}_{\mathrm{pCASL},\mathrm{init}}= \sum_{i=4}^{7}{\uptau }_{i}$$

$${\mathrm{SBD}}_{\mathrm{pCASL},\mathrm{new}}= {R}_{\mathrm{rel},1}\times {\mathrm{SBD}}_{\mathrm{pCASL},\mathrm{init}}$$

$${\uptau }_{7,\mathrm{ new}}=\frac{{\mathrm{SBD}}_{\mathrm{pCASL},\mathrm{new}}}{2}$$

$${\uptau }_{6,\mathrm{new}}=\frac{{\mathrm{SBD}}_{\mathrm{pCASL},\mathrm{new}}}{2}$$

$${\uptau }_{5,\mathrm{new}}=\frac{{{\mathrm{SBD}}_{\mathrm{pCASL},\mathrm{init}}-\mathrm{SBD}}_{\mathrm{pCASL},\mathrm{new}}}{2}$$

$${\uptau }_{4,\mathrm{new}}=\frac{{{\mathrm{SBD}}_{\mathrm{pCASL},\mathrm{init}}-\mathrm{SBD}}_{\mathrm{pCASL},\mathrm{new}}}{2}$$

$${\uptau }_{1-3,\mathrm{new}}= {\uptau }_{1-3,\mathrm{old}}$$

**Step 2:**

The first and third matrix rows (#1 and #3) can be used to decode another intermediate PWI (PWI#3) (outlined in purple). Depending on the state of perfusion at this TI, ${R}_{\mathrm{rel},2}<$ threshold (CASE 1) or $\ge$ threshold (CASE 2). In CASE 1, the brain is less perfused in PWI#3 than in PWI#1. Therefore, the acquisition of the following matrix rows continues with adapted SBDs depending on ${R}_{\mathrm{rel},2}$:

$${\mathrm{SBD}}_{\mathrm{pCASL},\mathrm{old}}= \uptau _{6,\mathrm{old}}+\uptau _{7,\mathrm{old}}$$

$${\uptau }_{7,\mathrm{ new}}= {\mathrm{SBD}}_{\mathrm{pCASL},\mathrm{old}} \times {R}_{\mathrm{rel},2}$$

$${\uptau }_{6,\mathrm{ new}}= {\mathrm{SBD}}_{\mathrm{pCASL},\mathrm{old}}- {\uptau }_{7,\mathrm{ new}}$$

$${\uptau }_{1-5,\mathrm{new}}= {\uptau }_{1-5,\mathrm{old}}$$

With ${\uptau }_{\mathrm{old}}$ = ${\uptau }_{\mathrm{new}}$ from Step 1.

Since the final duration of the pCASL subbolus is already defined in these matrix rows, all further matrix rows are acquired accordingly and the measurement is completed.

In CASE 2, the brain in PWI#3 is comparably perfused to PWI#1. To achieve the highest possible SNR in the final pCASL-subbolus, the third matrix row (#3) should be acquired again with appropriately adjusted SBDs. The new TI of the decodable PWI#3′ (yellow) lies exactly between the TI of PWI#2 and PWI#1.

**Step 3:**

In Step 3, the newly decoded PWI#3′ (yellow) is compared with the old PWI#3 (dark purple) from CASE 2 in Step 2. In CASE 1, PWI#3′ is less perfused than PWI#3, so the final pCASL-PLD should be between the two compared PWIs, again depending on the relative difference in perfusion.

In case 2, PWI#3′ shows a comparable perfusion status to the old PWI#3, suggesting that the final pCASL-subbolus might even be longer with a low probability of ATD artifacts. Since another repetition of matrix row #3 is not allowed to keep acquisition time as low as possible, the final pCASL-BD is acquired as long as possible, considering the minimum allowed SBD for the adjacent subbolus (${\tau }_{6}$).

A run-through of the algorithm with example start and end values for SBDs in the schematic of Fig. 2 can be found in Online Resource 1 Figure S3. The complete algorithm with calculations of all SBDs can be found in Online Resource 2.

## Methods

### Data acquisition

Five healthy volunteers (aged 21–57 years; one male) were scanned with a 3-Tesla MR-system (MAGNETOM Skyra, SIEMENS Healthineers AG, Erlangen, Germany) and a 20-channel head coil. Written informed consent was obtained before measurements, and the study was conducted according to a general protocol for the development of pulse sequences approved by the local ethics committee.

All subjects were scanned with a measurement protocol consisting of a three-dimensional T_1_-weighted gradient-echo acquisition (MPRAGE), time-of-flight (TOF), *M*_0_ and multiple ASL measurements performed in a single-shot (ss) and segmented (seg) acquisition scheme.

During acquisition, the intermediate PWIs were processed with the in-house developed processing program in the manufacturer’s own image calculation environment (ICE). The calculated adjustments for the SBD timings for the subsequent encoding matrix row were fed back to the sequence, where the SBDs were then adjusted during runtime.

### Time of flight angiography

For positioning the labeling slab accurately, a low-resolution TOF angiogram of the lower head and neck was acquired. The labeling plane was located as perpendicular to the carotid and vertebral arteries as anatomy allows, which is normally shortly upstream the V3-segment of the vertebral arteries. Sequence parameters were: field of view (FOV) = 200 × 150 × 76 mm^3^, matrix size: 256 × 192 × 76 (interpolated to 512 × 384 × 76), partial Fourier = 7/8, TR/TE = 21/3.48 ms, acquisition time (TA) = 1:02 min.

### 3D GRASE

For all image acquisitions for the ASL MR-scans an identical ss or seg three-dimensional (3D) gradient and spin echo (GRASE) readout was used [33]. Sequence parameters were: FOV = 256 × 192 × 96 mm^3^, matrix size: 64 × 48 × 24 (interpolated to 128 × 96 × 24), four segments (seg), turbo factor = 24 (ss), turbo factor = 12 (seg), echo planar imaging factor = 48 (ss), echo planar imaging factor = 24 (seg), receiver bandwidth = 2298 Hz/pixel, flip angle (refocusing pulse) = 120°, TE = 29.5 ms (ss), TE = 18 ms (seg).

### *M*_0_ images

For quantification purposes, three M_0_ images with different TIs [400, 1700, 3000] ms were acquired. Images were acquired using a slab-selective saturation recovery method with the same 3D GRASE reading procedure used for the WH-pCASL measurements.

### Background suppression

For all ASL acquisitions, background suppression was used. After a saturation module, two hyperbolic secant pulses (ß = 800 s^−1^, µ = 24.0) [34] were applied during the encoding phase (compare Fig. 1C). The timings θ_1_ and θ_2_ of the two inversion pulses were calculated using an analytical solution for nulling components with relaxation times T1_opt_ = 700 ms and 2 × T1_opt_ = 1400 ms [33]. The label/control condition was inverted after every inversion pulse as described in [24]. Prior to the 3D GRASE readout, tissue signal outside the imaging slab is nulled with multiple modulated saturation pulses [33].

### Fixed WH-pCASL

The 8 × 8 Walsh-ordered Hadamard matrix [28] (Fig. 1A) was used as an encoding scheme. For these acquisitions with fixed predefined SBDs, the different scenarios are listed in Table 1. The timing with intentionally too-long pCASL-BD/too-short pCASL-PLD for healthy subjects was chosen to simulate ATD artifacts in the pCASL-subbolus PWI. With TR = 5000 ms and one preparation scan, respectively, the durations for these acquisitions were TA = 0:45 min (ss) and TA = 2:45 min (seg).

**Table 1:** Different timing scenarios for standard WH-pCASL and the start values for adaptive WH-pCASL

Fixed WH-pCASL (ss and seg)				
	Max TI [ms]	pCASL-BD ${\tau }_{7}$ [ms]	pCASL-PLD [ms]	SBDs [ms] (subbolus ${\tau }_{6-1}$)
Recommended pCASL-PLD/pCASL-BD*	3600	1800	1800	(500, 240, 240, 240, 240, 240)
pCASL-BD too short/pCASL-PLD too long	3600	1000	2600	(500, 400, 400, 400, 400, 400)
pCASL-BD too long/pCASL-PLD too short	3600	2600	1000	(150, 150, 150, 150, 150, 150)

Adaptive WH-pCASL (ss and seg)				
	Max TI [ms]	pCASL-BD [ms]	pCASL-PLD [ms]	SBDs [ms] (subbolus ${\tau }_{7-1}$)
Start values	3600	To be estimated during runtime	To be estimated during runtime	(695, 695, 695, 695, 240, 240, 240)

*ss* single-shot, *seg* segmented, *PLD* post labeling delay, *TI* inflow time, *BD* bolus duration, *SBD* subbolus duration

^*^White paper recommendation for healthy subjects aged < 70 years [15] and with T1 relaxation adapted BDs after[21]

### Adaptive WH-pCASL

The proposed adaptive 8 × 8 Walsh-ordered matrix with an additional help encoding row (Fig. 1A) was used as an encoding scheme.

Three repeated measurements were performed for each of the adaptive WH-pCASL acquisitions to test the robustness of the algorithm. Each repetition had the same starting values and the algorithm adjusted the imaging timings, respectively. For the adaptive measurements that were compared with the quantification results of the measurements with fixed SBDs, the initial duration of each subbolus is listed in Table 1. The first two rows of the encoding matrix are acquired with these values. As additional encoding rows are acquired, the SBDs are adjusted accordingly. With TR = 5000 ms and one preparation scan, respectively, the durations for these acquisitions were TA = 0:50 min (+ 5 s if one encoding row has to be repeated) (ss) and TA = 3:05 min (+ 20 s if one encoding row has to be repeated) (seg).

In addition, the behavior of the algorithm presented here was tested with changed start values for PLD_1_ and max TI’s for ss only (compare Online Resource 1 Table S1). With TR = 5500 ms and one preparation scan, each TA = 0:55 min (+ 5.5 s if one encoded row is repeated).

### Adaption constraints

The threshold for the relative difference in NoV (*R*_rel_), which determines whether the pCASL-BD must be shortened or lengthened, was set at 10%. Experimentally, this number has proven to be appropriate.

Since the signal of the subboli depends on T_1_ relaxation effects, subboli with a longer TI lose more signal than boli with a shorter TI. Therefore, certain minimum allowable SBDs were defined in the algorithm for each subbolus. For this work, the minimum allowed pCASL-BD (${\tau }_{7}$) was set to 1000 ms, the minimum allowed SBD for the subbolus (${\tau }_{6}$) before the pCASL-subbolus was limited to 300 ms, and the next two subboli (${\tau }_{5-4}$) had to have a minimum duration of 200 ms. In addition, to keep the total scan time low and the measurement as efficient as possible, only one repeated acquisition of encoding row #3 was allowed.

### Image analysis

#### Evaluation of the robustness of the adaption algorithm

The final adjusted SBDs and pCASL-BDs/PLDs from the three repeated measurements, each performed in the ss- and seg-data acquisition schemes were compared within-subject and between-subjects. Standard deviations within-subject and between-subjects (even when the number of subjects and repeats was relatively small) were used as the method of evaluation. A one-way ANOVA test was performed to quantify the inter-subject dependency.

In addition, the pCASL-subbolus PWI and the preceding PWI from subbolus ${\tau }_{6}$ were visually inspected to determine whether in this PWI the brain was already completely filled with labeled blood or whether, for instance, the outer brain areas were not yet perfused.

#### Calculation of CBF and ATT maps

The decoded PWIs were processed using the Oxford Centre for Functional MRI of the Brain (FMRIB) software library (FSL) [35]. Structural data were processed using the fsl_anat pipeline. Whole-brain masks and gray and white matter probability maps (GM, WM) were used to mask the voxels of interest. Voxel-wise fitting was performed within the whole-brain mask using the underlying two-compartment model in FSL. *M*_0_ map was calculated by applying a monoexponential non-linear least squares fit using the Python function curve_fit from the Scipy.Optimize library. The result was fed into the FSL quantification pipeline. The calculated mean CBF and ATT values with their corresponding standard deviations as an indicator of the uncertainty of the performed fit were then compared for GM and WM with the different WH-pCASL timings and the adaptive WH-pCASL results.

#### Analysis of ATD artifacts

The final pCASL-subbolus PWIs with different PLDs were visually examined for hyperintense regions as signs of macrovascular signal. In addition, the mean and median of the image histograms were determined and compared between the different timings. The higher the difference, the more “outliers” of voxel signal values that could indicate hyperintense signal and thus the presence of ATD artifacts presumably exist.

In the measurements with a pCASL-PLD_7_ = 1000 ms, ATD artifacts are likely to be present in healthy subjects [36]. The distance between mean and median in the histogram of these images can therefore serve as a landmark for the results with other timings.

## Results

Table 2 shows the resulting pCASL-subbolus PLDs for all five volunteers and three repetitions of the same measurement. The mean standard deviations of the final intra-subject pCASL-PLD_7_ are 38.5 ms (ss) and 36.33 ms (seg). In comparison, the mean standard deviation of the final inter-subjects pCASL-PLD_7_ are 122.87 ms (ss) and 127.87 ms (seg). The mean difference between adaption results for pCASL-PLD_7_ from ss and seg acquisition is 50.47 $\pm$ 9.84 ms.

**Table 2:** Adapted pCASL-subbolus post labeling delay (PLD) [ms] (all subjects)

Subject	1	2	3	4	5	
Max TI = 3600 ms	start PLD = 100 ms					
Single-shot acquisition adapted pCASL-subbolus PLD [ms]						
Rep 0	1781	1520	1807	1833	1898	
Rep 1	1731	1520	1824	1815	1927	
Rep 2	1757	1636	1639	1787	1893	
Mean	**1756.33**	**1558.67**	**1756.67**	**1811.67**	**1906.00**	
SD	**20.42**	**54.68**	**83.49**	**18.93**	**14.99**	
p (one-way ANOVA)/Mean SD intra-subject	< 0.001					38.5
SD inter-subjects (all repetitions)						**122.87**
Segmented acquisition adapted pCASL-subbolus PLD [ms]						
Rep 0	1816	1520	1871	1757	1808	
Rep 1	1877	1520	1800	1753	1830	
Rep 2	1748	1520	1736	1727	1946	
Mean	**1813.67**	**1520.00**	**1802.33**	**1745.67**	**1861.33**	
SD	**52.69**	**0.00**	**55.14**	**13.3**	**60.54**	
p (one-way ANOVA)/Mean SD intra-subject	< 0.001					36.33
SD inter-subjects (all repetitions)						**127.87**
Absolute difference single-shot—segmented [ms]						
Single shot mean	1756.33	1558.67	1756.67	1811.67	1906.00	
Segmented mean	1813.67	1520.00	1802.33	1745.67	1861.33	
Absolute difference	**57.33**	**38.67**	**45.67**	**66.00**	**44.67**	
Mean difference						50.47
SD						**9.84**

For derived values, i.e. bold values in last column and 3rd last row, statistical testing is not beneficial

For 3 of 5 subjects, the algorithm repeated the acquisition of matrix encoding row #3 once per measurement.

**Fig. 3:**
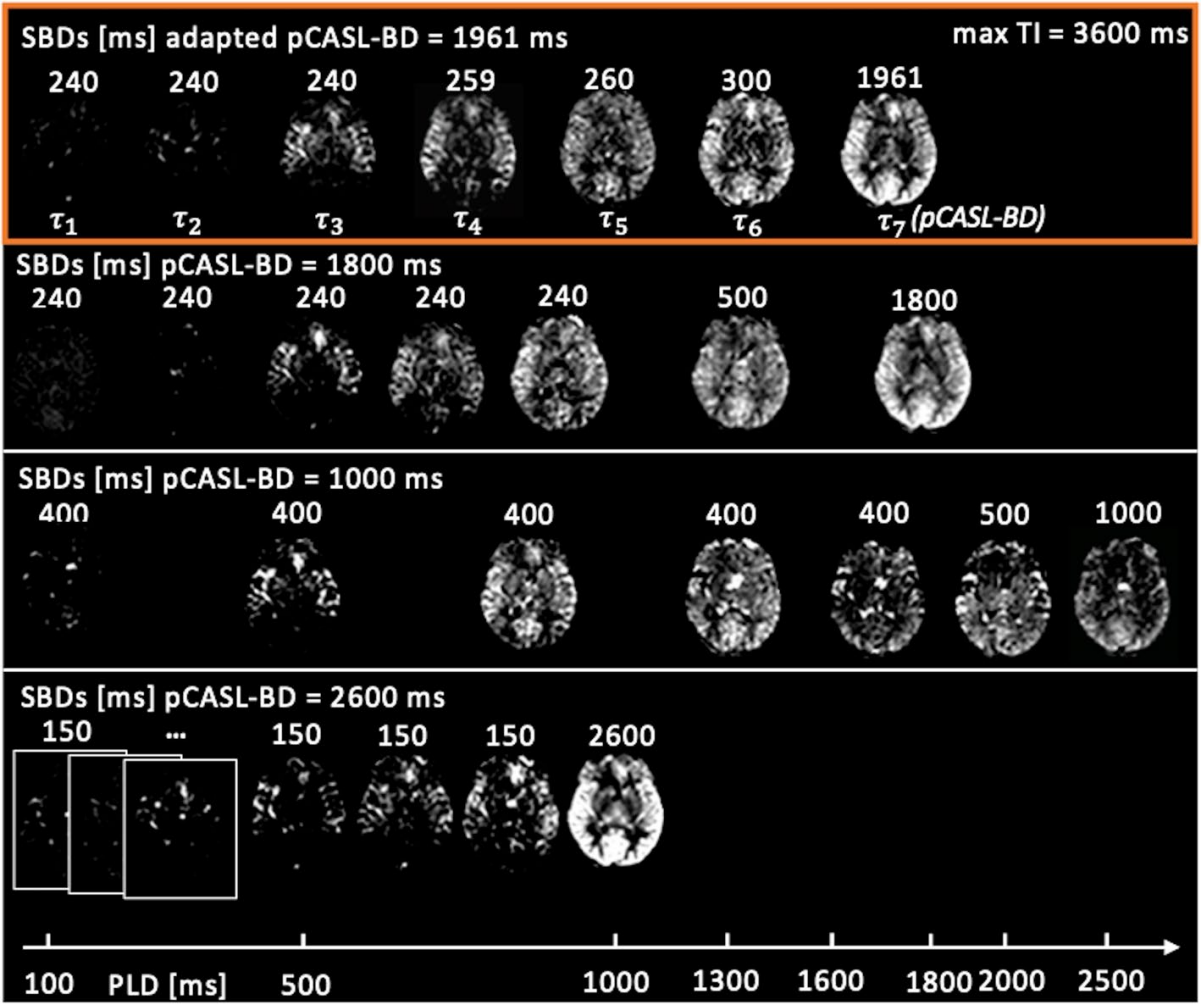
Comparing four different timings, the resulting sampling of the inflow of labeled blood for one subject is shown. Each timing option (from top to bottom): (1) automatically adapted pCASL bolus duration (BD) (adapted pCASL-BD = 1961 ms), (2) pCASL-BD = 1800 ms (recommended [15, 23]), (3) pCASL-BD = 1000 ms (intentionally too short), (4) pCASL-BD = 2600 ms (intentionally too long) has different effective bolus post labeling delays (PLD) and subbolus durations (SBD) (${\uptau }_{1-7}$)

Figure 3 compares an example adaption result with the recommended [15, 21] but not optimized and non-optimal timings for healthy subjects. On visual inspection, it can be seen that timing with the pCASL-BD = 1000 ms has a low signal and the brain is already fully perfused in early subboli. Timing with the pCASL-BD = 2600 ms shows an insufficient sampling of the early subbolus and hyperintense signal in the pCASL-subbolus PWI.

**Fig. 4:**
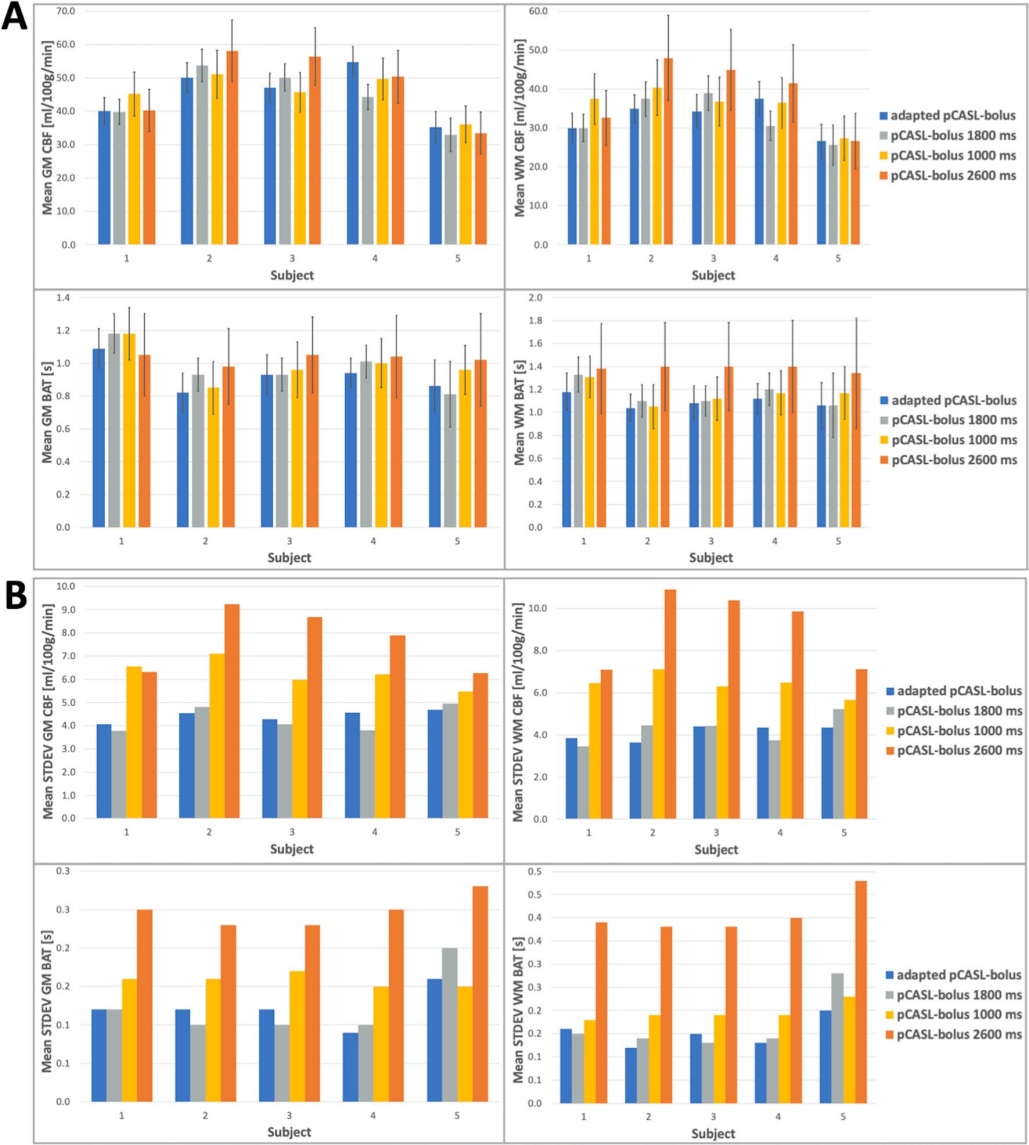
**A** The mean quantification results for CBF and ATT in the gray and white matter (GM, WM) from the single-shot acquisitions are shown for the four different timing options and all subjects. **B** The respective mean standard deviations as a measure of uncertainty in the quantification results are plotted side-by-side to compare the influence of sampling on confidence in the quantification results. The final adjusted pCASL-subbolus durations from the adaption process are: 1843 ms (subject 1), 1964 ms (subject 2), 1961 ms (subject 3), 1813 ms (subject 4), 1673 ms (subject 5)

**Fig. 5:**
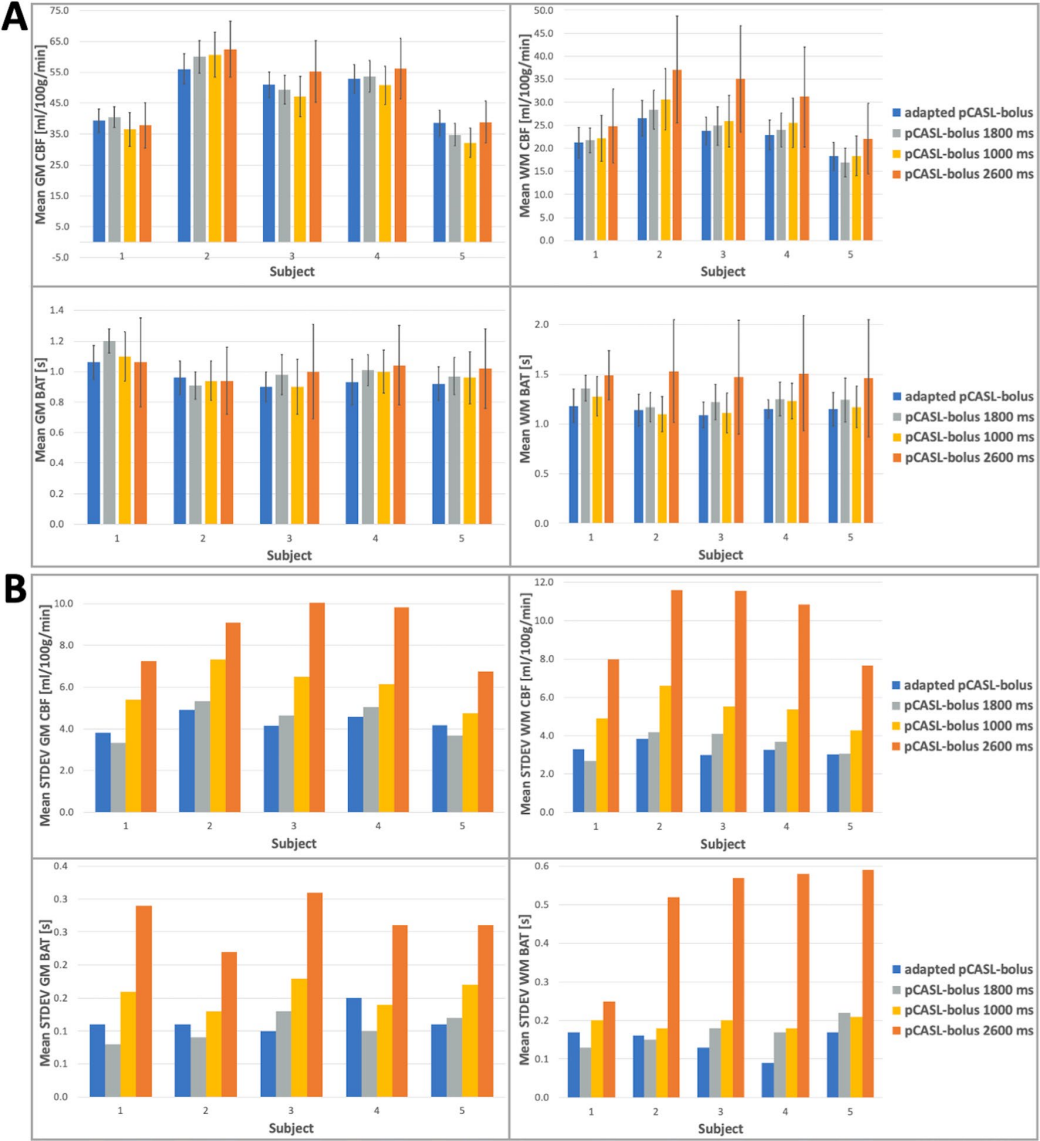
**A** Same as in Fig. 4, but here the mean quantification results for CBF and ATT in the gray and white matter (GM, WM) from the segmented acquisitions are shown for the four different timing options and all subjects. **B** The respective mean standard deviations as a measure of uncertainty in the quantification results are plotted side-by-side to compare the influence of sampling on confidence in the quantification results. The final adapted pCASL bolus durations from the adaption process are: 1852 ms (subject 1), 2080 ms (subject 2),1864 ms (subject 3), 1843 ms (subject 4), 1654 ms (subject 5)

Figure 4A shows the mean CBF and ATT results of all ss-experiments in GM and WM with their associated standard deviations. For illustration purposes, the standard deviations can be directly compared in Fig. 4B. The same is shown for all experiments with the seg-acquisition scheme in Fig. 5A, B.

**Fig. 6:**
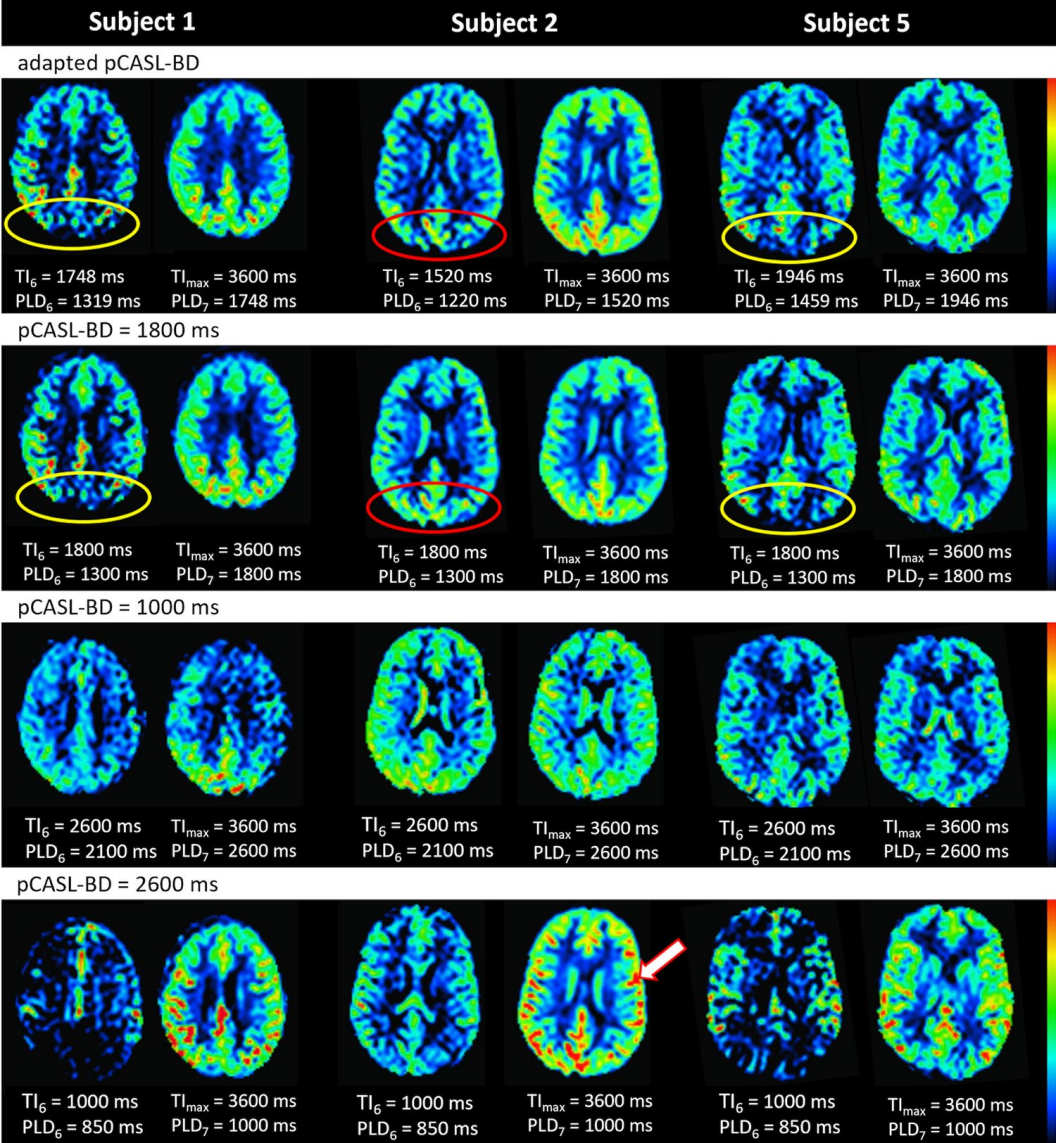
Shown are the perfusion-weighted images (PWI) of the long pCASL-subbolus (right) and the preceding subbolus (left) of three of the five subjects. The first row shows the results of the adaption, the second shows the recommended timing with pCASL bolus duration (BD) = 1800 ms, and the third and fourth show the suboptimal timing with pCASL-BD = 1000 ms and pCASL-BD = 2600 ms, respectively. With the adjusted timing, the brain is already almost completely perfused at the inflow time (TI) before the pCASL-subbolus, except for the outer brain region (see yellow and red circles). The recommended timing was also optimal for subjects 1 and 5, but not ideal for subject 2 (red circle), where the brain was already fully perfused at TI_6_. The third row shows a fully perfused brain at both TIs. The fourth row shows hyperintense signals in the pCASL-subbolus PWI, indicating the presence of intravascular signal

In Fig. 6, the pCASL-subbolus PWI can be compared visually with the preceeding PWI in three of five subjects. The focus is on the perfusion status in the outer brain regions. It also allows visual inspection and comparison of potential ATD artifacts in suboptimal timings (e.g., arrow in PWI pCASL-BD = 2600 ms).

**Fig. 7:**
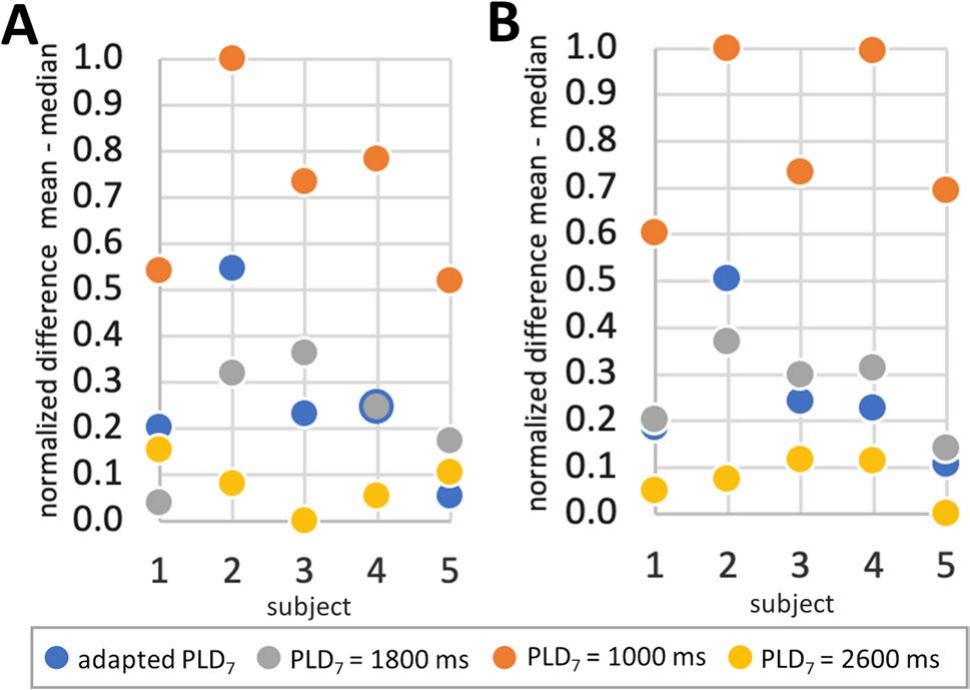
The normalized difference between the mean and median values obtained from the histograms of the perfusion-weighted images decoded from the pCASL-subbolus are compared for all timings and subjects as an indicator of the presence of hyperintense signals. **A** Results of single-shot acquisition, **B** results of segmented acquisition

The quantitative results of a preliminary analysis for ATD artifacts are compared in Fig. 7. For this purpose, a normalized difference between the mean value of the pCASL-subbolus PWI histogram and the median value is calculated for all timings and subjects.

**Fig. 8:**
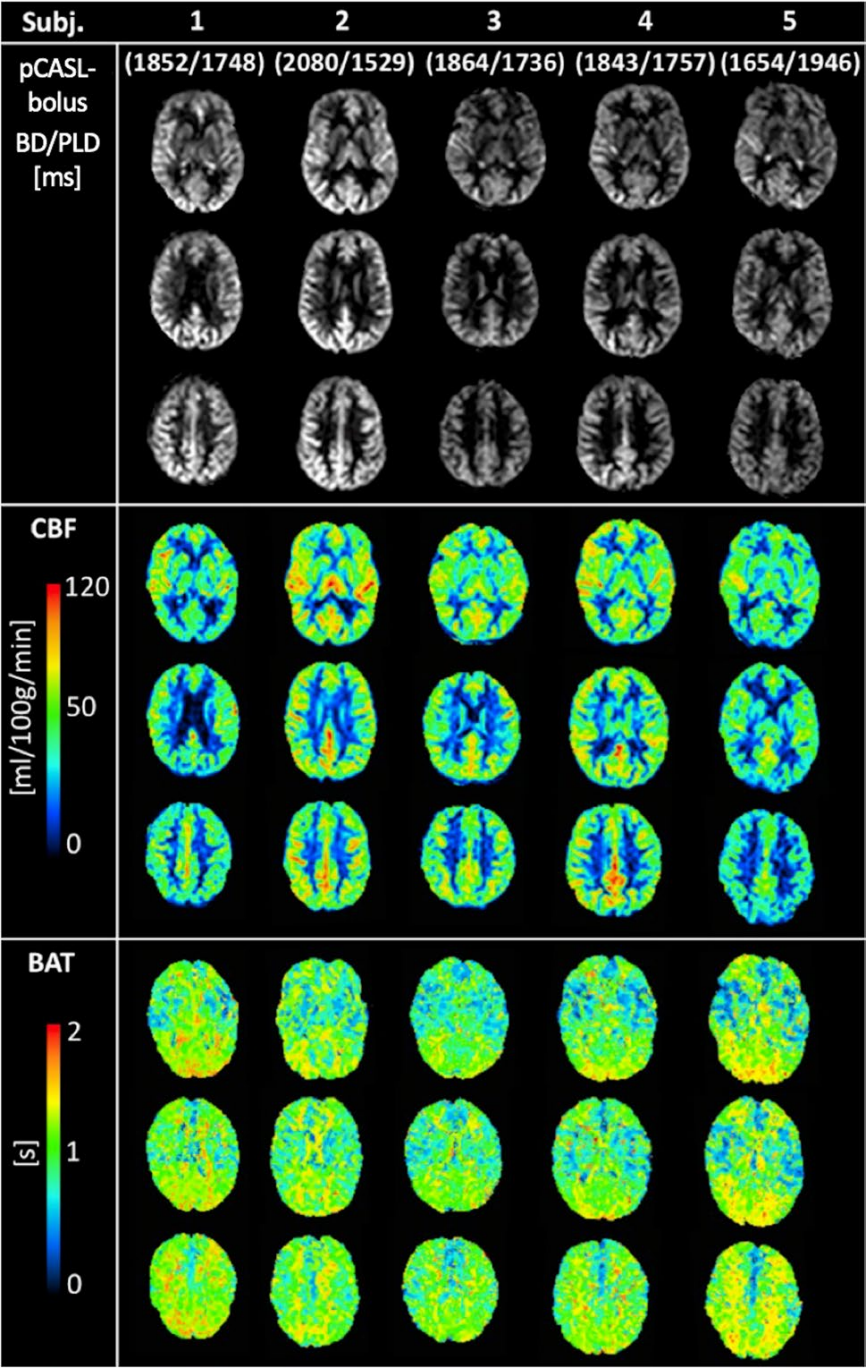
Results of adapted timing with respective pCASL bolus duration (BD) and its effective post-labeling delay (PLD) for all subjects. Three out of 24 slices from the perfusion-weighted images, the respective CBF maps [ml/100 g/min] and the ATT maps [s]

Finally, Fig. 8 shows the PWI (three out of 24 slices) for the adapted pCASL-PLD_7_ and the corresponding CBF and ATT maps.

Additionally, in Online Resource 1 Table S2 the adjustment results for all subjects with different starting values of PLDs and TI_max_ can be found. At TI_max_ = 4600 ms and a start PLD_1_ of 100 ms, the final pCASL-PLD_7_ was set exactly or close to its minimum allowed duration within the predefined constraints for SBDs (see “Methods” section). The same is true for the next setting with a start PLD_1_ of 1100 ms. For the measurements with TI_max_ = 2600 ms, the maximum possible pCASL-PLD_7_ would have been 1600 ms. The algorithm ended here in one of the three repetitions in subject 1 and was closed once in subject 5. All other results ended at the minimum allowed PLD_7_ of 1520 ms.

## Discussion

In this paper, a possible technical solution for subject-specific adjustment of SBDs during the runtime of a measurement with acceptable time loss is presented. The technical feasibility is evaluated to possibly find an optimized pCASL-BD/PLD for presumptive avoidance of ATD artifacts in time-encoded ASL measurements. The method has not yet been tested in patients, therefore no statement can be made about an actual reduction of ATD artifacts in the case of prolonged ATTs.

### Evaluation of the robustness of the adaption algorithm

The proposed time-encoded ASL adaption algorithm consistently found individual timings for the SBDs and pCASL-BDs. In the three repetitions, a relatively low intra-subject standard deviation can be observed, which suggests a robust performance and good repeatability of the proposed approach. Additionally, the standard deviation within a subject was smaller than the standard deviation between subjects for both ss- and seg-acquisition (see Table 1). This may indicate that the algorithm adjusts the SBDs according to individual inflow behavior, which varies among healthy subjects in general and changes with gender and age [11, 13, 15, 16, 37]. The algorithm logically adjusted the SBDs differently for each subject but within the range of expected normal inflow behavior in healthy subjects. Except for subject 2, the adjusted pCASL-PLD_7_ values were all within the range of recommended times for healthy subjects aged < 70 years [15, 21]. For subject 2, the algorithm chose the smallest pCASL-PLD_7_ that was possible within the pre-set adaption restraints. Figure 6 (red circle) shows that the brain was indeed already almost completely filled at the TI before the pCASL-subbolus. In comparison, the TI before the recommended pCASL-PLD_7_ (= 1800 ms) already shows a well-filled brain, even in the outer brain regions. Therefore, the results suggest that subject 2 indeed had a faster inflow response than the other four subjects.

The measurements in the ss-acquisition scheme have a much shorter scan time than the segmented measurements. However, the image quality is inherently lower. It is also noted that the mean difference between the adaption results for pCASL-PLD_7_ between the two schemes is higher than the measured standard deviation between three repetitions within a subject. However, the short measurement time of the ss-data could be advantageous in routine clinical practice or serve as a calibration measurement before another, possibly more advanced ASL measurement like a multi-TE measurement to determine the integrity of the blood–brain barrier [38]. In this case, even lower resolution would be conceivable because the calibration perfusion data would not subsequently be used for quality determination and diagnosis.

When different starting values were selected for PLD_1_ and TI_max_ (see Online Resource 1 Table S1), the algorithm adjusted the SBDs and the pCASL-PLD_7_ accordingly within the predefined constraints on the minimum allowable SBDs (compare Online Resource 1 Table S2). Thus, with a longer TI_max_ of 4600 ms and a relatively late TI_3_ of 1300 ms, the algorithm consistently adjusted the pCASL-PLD_7_ to the minimum allowable value of 2000 ms, except for subject 1, indicating that the algorithm automatically recognized that the brain was perfused at an earlier TI and therefore adjusted the pCASL-subbolus to the maximum possible duration to obtain the highest possible SNR. TI_3_ defines the inflow time of the second decoded PWI (outlined in green in Fig. 2). Since the SBDs ${\uptau }_{1-3}$ cannot be changed during the measurement, TI_3_ is also not changeable during the measurement. As all subjects showed a normal inflow behavior, a late TI_3_ is therefore not optimal, because here the brain is already perfused to a large extent. Thus, these experiments only served to show that the algorithm reacts accordingly to a change in the start values.

The same is true for the second setting (here TI_3_ = 1820 ms). Again, the algorithm set the pCASL-PLD_7_ to the smallest possible value in all subjects. In contrast, a TI_max_ of 2600 ms and TI_3_ = 820 ms should have resulted in a maximum allowable pCASL-PLD_7_ of 1600 ms. This was the case only for subject 1 in one of three repetitions. Instead, the algorithm brought the pCASL-PLD_7_ back to the minimum allowed value of 1520 ms in almost all repetitions and subjects. A plausible reason for this could be that only a small range of 80 ms between the maximum and minimum allowed pCASL-PLD_7_ could be determined anyway. Therefore, the range may have simply been too small to reliably find the optimal pCASL-PLD_7_. For comparison, in the selected start setting with TI_max_ = 3600 ms, the pCASL-PLD_7_ could be adapted in a range of 1080 ms.

To further test the robustness and behavior of the proposed algorithm, the constraints for SBDs and pCASL-BD should be adjusted according to the studied start settings to allow for a wider range of adaption. Furthermore, the results of the algorithm could be simulated in future work with artificial PWIs to detect unusual behavior.

### Evaluation of CBF and ATT maps

The quantification results for mean CBF and ATT can be retrieved from Figs. 4, 5.

It can be recognized that the CBF values vary slightly between the ss- and seg-data. For GM in the range of the determined standard deviation, but for WM in a larger degree. The values of ATT, on the other hand, varied almost exclusively in the range of the determined uncertainties. However, more interesting for the evaluation of the algorithm presented here is the difference between the mean CBF and ATT with the respective standard deviation as a measure of the uncertainty of the fit for the different timings. For all subjects, the standard deviations of the adapted timing are within the range of the standard deviations from the recommended timing for healthy humans, while the uncertainty in suboptimal sampling increases significantly especially for the intentionally too-short pCASL-PLD of 1000 ms (pCASL-BD = 2600 ms). This indicates that especially an improper sampling of the inflow curve and a final pCASL-subbolus PWI with ATD artifacts can lead to a poor fit result.

### Analysis of ATD artifacts

As desired, the brain was almost completely perfused in the PWI before the pCASL-PWI (Fig. 6). On visual inspection, the pCASL-PWI of the adapted timing also show no signs of ATD artifacts, except that the results from subject 2 show a hyperintense signal in the outer brain area. This could be due to the rather short pCASL-PLD_7_ in this subject.

In the results of the intentionally suboptimal timing with pCASL-BD = 2600 ms, hyperintense signal areas are clearly visible in all subjects. Analysis of the image histograms also shows that the difference between mean and median is larger for this suboptimal timing than for the other three timings studied (Fig. 7). Because only healthy subjects were measured in this study, the adjusted data and the data with the recommended pCASL-BD show similar results. On average, the adapted data show a slightly smaller difference, which is mainly due to the fact that the adapted pCASL-PLD_7_ is slightly longer than 1800 ms for subjects 3, 4, and 5. In contrast, the difference between mean and median is larger for subject 2. Here, the algorithm detected a low pCASL-PLD_7_ of 1520 ms and one can visually see some hyperintense signals in the outer brain area (Fig. 6). However, Fig. 6 also shows that a pCASL-PLD_7_ of 1800 ms (pCASL-BD = 1800 ms) would have been too long for subject 2, as the brain seems to be already fully perfused on the PWI before the pCASL-PWI (indicated by red cycle). Therefore, an optimal timing is probably slightly above the determined 1520 ms, at least for the watershed regions. The algorithm consistently determined the minimum possible pCASL-PLD_7_ in subject 2. The optimal, slightly longer PLD_7_ was probably not found because of the minimum allowable SBD ${\tau }_{6}$.

The difference between the mean and median of the image histogram is even smaller for the second suboptimal timing (pCASL-BD = 1000 ms) than for all other timings (see Fig. 7). This is due to the relatively long pCASL-PLD_7_ in healthy subjects and the relatively short BD, which also leads to a lower SNR.

### Limitations, optimizations and future applications

The most important limitation of the present study is that only healthy volunteers were scanned. However, this study was designed to test and present the technical solution such an adaptive approach could provide when individual sequence timing adaptions are desired. To assess the clinical value of the adaptive WH-pCASL, studies should be performed in different age groups and in patients. The small number of subjects is sufficient for a technical feasibility study such as the presented.

Moreover, for a detailed statistical analysis of the robustness of the algorithm, more repetitions should be measured in a test–retest design. The algorithm could also be challenged as mentioned by feeding it with different simulated input data. Preliminary tests have shown a robust behavior, but this was not yet studied in detail.

Furthermore, the decision criteria could be optimized or combined with other parameters such as entropy for instance, to find potential ATD artifacts independently of the here presented “number of filled voxels above noise level” rule. Another point of discussion in this context would be the defined threshold as of when a voxel is considered “filled” and when it is not.

In the current implementation of the presented method, the relative difference in NoV between two compared PWIs directly determines the shortening or lengthening of pCASL-BD and SBDs within the predefined constraints. Whether this adjustment could also be based on a different rule will be analyzed in future applications. The predefined SBD constraints could also be modified, although, as in this study, T_1_ relaxation and SNR constraints when SBDs become too short should always be kept in mind.

In addition to the adjustments of the SBD constraints or the decision criteria when and how much to adjust the SBDs for each acquired matrix row, the presented algorithm offers further theoretical strategies to make the best use of its flexibility. One possible adaptation would be to consider a desired “filling level” of the brain in the intermediate PWIs. At the expense of measurement time, since individual matrix rows would have to be acquired repeatedly more often, a flexible number of iterations at each decision level would be conceivable until a certain “filling level” of the brain is reached at each step.

Motion is a regular problem in time-encoded pCASL and can affect the whole measurement. The method presented does not protect against this and motion artefacts could potentially harm the timing adjustment process. One advantage of the presented approach is that only two rows of the encoding matrix are used to calculate the intermediate PWI in each case. However, patient movement that occurs between these two image acquisitions can corrupt the intermediate PWIs and the final decoding of all PWIs as well. Therefore, it is suggested to perform on-going constituency checks throughout the measurement and a retrospective motion correction before each calculation of the individual intermediate PWIs. Once all matrix rows have been acquired, retrospective motion correction can then be performed again using either the encoded image of the first matrix row or the M0 image as a reference image for registering all encoded images before decoding the final PWIs. Both methods already work within the implemented online image reconstruction pipeline but were not necessary in this work because the registered motion of the subjects was very low. If too much motion of the images was noticed during image registration, the described method could also be used to trigger a new acquisition of the last matrix row(s). Other prospective motion correction methods without [39] or with external devices [40] might also be applicable in the future.

## Conclusion

The proposed adaptation algorithm for FL WH-pCASL is able to successfully calculate intermediate PWI and adjust sequence timing during active scanning based on individual inflow behavior of labeled blood in time-encoded ASL imaging. The presented acquisition strategy thus allows subject-specific adjustments within predefined limits of ASL parameters such as BD and PLD during an ongoing measurement. In this way, time-encoded ASL can be automatically tailored to an individual subject. The presented method aims to reduce ATD artifacts, improve ASL perfusion quantification results, and avoid reacquisitions in clinical settings. However, the extent to which the results are transferable to patients and provide benefit needs to be investigated in follow-up studies.

### Supplementary Information

Below is the link to the electronic supplementary material.

Supplementary file1 (PDF 1074 KB)

Supplementary file2 (PDF 994 KB)

## Data Availability

Upon individual requests, the authors will provide access to the data used accordingly.
