# Supplementary material for: Subject-specific timing adaption in time-encoded arterial spin labeling imaging
Source: MAGMA. 2023 Sep 28;37(1):53–68. doi: 10.1007/s10334-023-01121-y (PMC10876770; doi:10.1007/s10334-023-01121-y)
Supplement: Supplementary file 1 — Supplementary file1 (PDF 1074 KB) [file 10334_2023_1121_MOESM1_ESM.pdf]

Supporting Information

Subject-Specific Timing Adaption in Time-Encoded Arterial Spin Labeling Imaging

Dr. rer. nat. Nora-Josefin Breutigam[1], Dr. rer. nat. Daniel Christopher Hoinkiss[1], Dr. rer. nat. Simon Konstandin[1,2], Mareike Alicja Buck[1,3] MSc, Amnah Mahroo[1] MSc, Prof. Dr. rer. nat. Klaus Eickel[1,2,4], Dr. rer. nat. Federico von Samson-Himmelstjerna[1,3], Prof. Dr. rer. nat. Matthias Günther[1,2,3]

[1]Imaging Physics, Fraunhofer Institute for Digital Medicine MEVIS, Bremen, Germany
[2]mediri GmbH, Heidelberg, Germany
[3]Faculty 1 (Physics/Electrical Engineering), University of Bremen, Bremen, Germany
[4]Bremerhaven University of Applied Science, Bremerhaven, Germany

Contact: nora-josefin.breutigam@mevis.fraunhofer.de

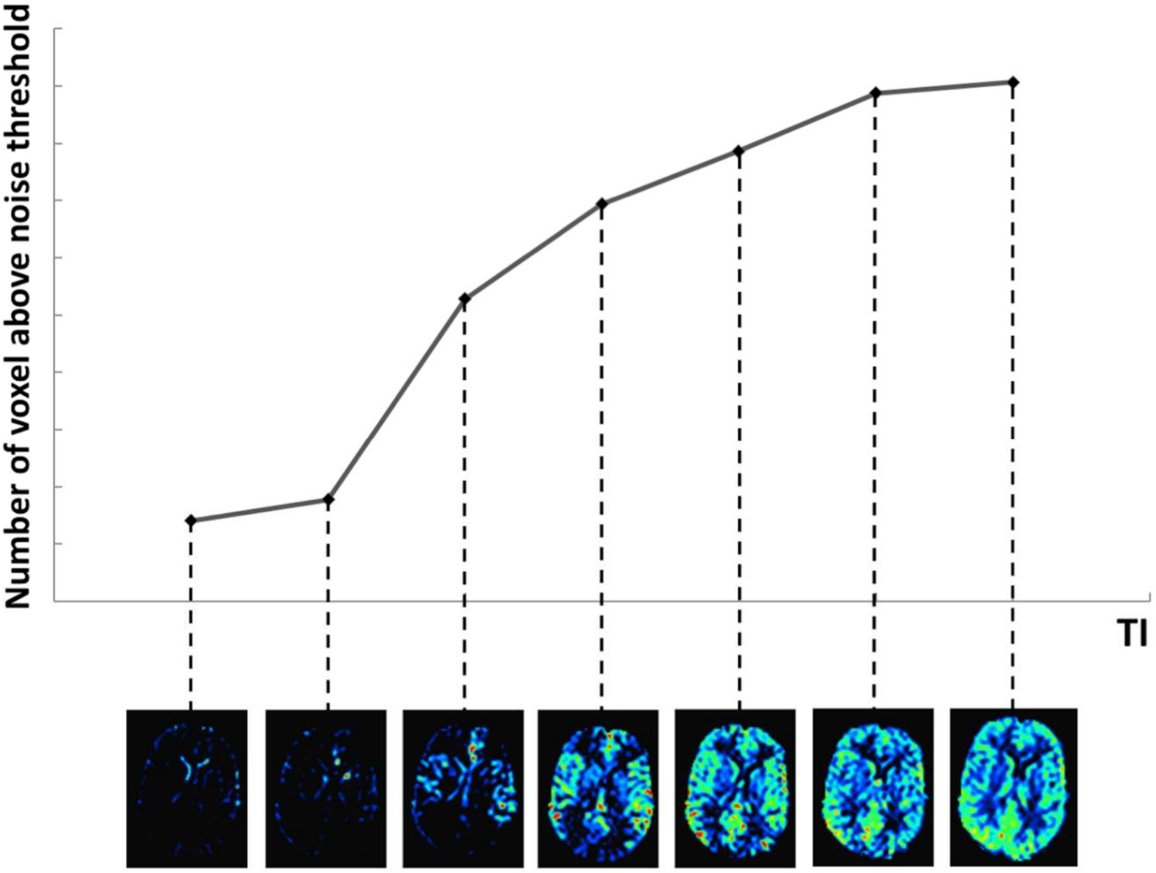

Figure S1: Example illustration of increasing number of voxels (NoV) with signal values above noise threshold [arbitrary unit] following the inflow times (TI) [ms] (here TI_{max} = 3600 ms).

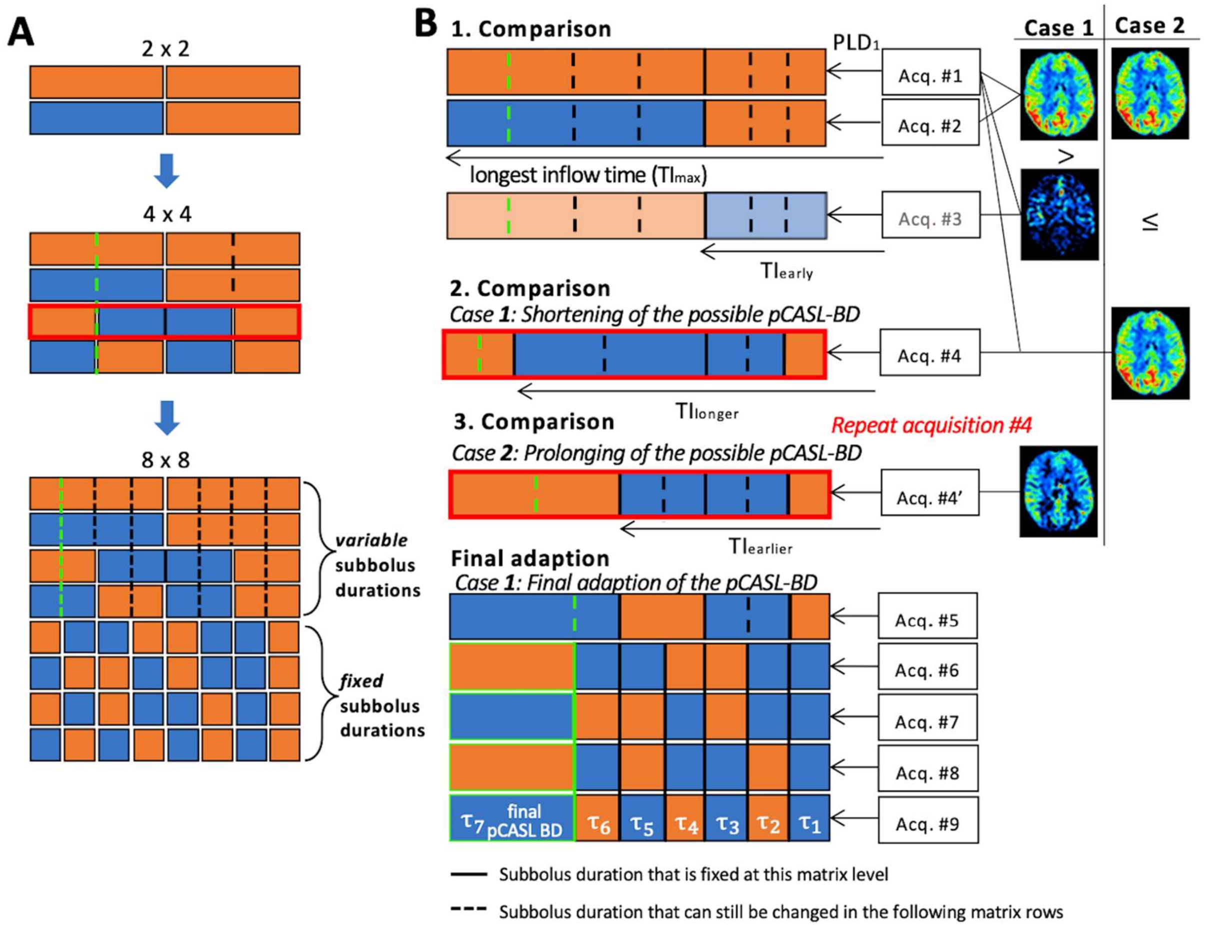

Figure S2: (A) The temporal evolution of the encoding matrix reveals the possibility of SBDs adaption during runtime. Square matrices of different sizes are effectively applied within one measurement. A 2x2 encoding matrix is applied after two image acquisitions, a 4x4 after four acquisitions, and an 8x8 after eight acquisitions. Virtual subboli are included in each matrix sequence whenever the labeling and control phases do not change (indicated with dashed line). The actual boundary of this virtual subbolus is not determined until the next row is acquired. Therefore, the first four matrix encoding rows in the final 8x8 matrix have variable SBDs at the beginning of the measurement. Once the durations are determined, the last four matrix rows have invariant SBDs until the acquisition is complete. (Based on [30]).

(B) A representative adaption run using the developed algorithm shows how the pCASL-subbolus duration (BD) adjustment is performed during the temporal evolution of the adaptive Walsh-ordered encoding matrix. The first intermediate perfusion-weighted images (PWI) can be retrieved from the initial 2x2 accuqisition (Acq. #1 and #2) and using the help encoding row (Acq. #3). The initial PWI is from the bolus with the longest inflow time (TI_{max}), while the PWI decoded from Acq. #1 and #3 represents an early TI_{early}. The number of voxels above the noise threshold (NoV) is counted and compared in a complete image volume of the PWIs. If the first case is true, the partial subbolus (blue subboli in Acq. #4) that encodes the next decoded PWI must have a longer TI_{longer}. In the next decoding step, the difference between NoV from PWI Acq. #1 and #4 and PWI from Acq. #1 and #2 is < threshold (case 2), which means that Acq. #4 must be repeated with an earlier $TI_{earlier}$ of the encoded "blue" partial subbolus. The next comparison from Acq. #4' shows that the brain is almost completely filled with blood, but not as much as in Acq. #4. Therefore, the final pCASL-PLD which defines the pCASL-BD must be between $TI_{earlier}$ from PWI Acq. #4' and TI_{longer} of PWI Acq. #4.

Table S1: Different start values for adaptive single-shot-WH-pCASL

		**adaptive WH-pCASL** (ss)			
		max TI [ms]	*pCASL-BD [ms]*	*pCASL-PLD [ms]*	*SBDs [ms] (subbolus τ_{7-1})*
start values	**A**	4600	To be estimated during runtime	To be estimated during runtime	(825, 825, 825, 825, 400, 400, 400)
	**B**	4600	To be estimated during runtime	To be estimated during runtime	(695, 695, 695, 695, 240, 240, 240)
	**C**	2600	To be estimated during runtime	To be estimated during runtime	(445, 445, 445, 445, 240, 240, 240)

single-shot (ss), post labeling delay (PLD), inflow time (TI), bolus duration (BD), subbolus duration (SBD)

Table S2: Adapted single-shot-WH-pCASL-subbolus post labeling delay (PLD) [ms] (all subjects)

**Results single-shot acquisition adapted pCASL-subbolus PLD [ms]**					
**Subject**	**1**	**2**	**3**	**4**	**5**
**A** *max TI = 4600 ms*	*start PLD = 100 ms*				
rep 0	2232	2000	2000	2000	2000
rep 1	2186	2000	2000	2000	2000
rep 2	2118	2000	2000	2000	2000
**mean**	**2178.67**	**2000.00**	**2000.00**	**2000.00**	**2000.00**
stdev	46.83	0.00	0.00	0.00	0.00
**B** *max TI = 4600 ms*	*start PLD = 1100 ms*				
rep 0	2520	2520	2520	2520	2520
rep 1	2520	2520	2520	2520	2520
rep 2	2520	2520	2520	2520	2520
**mean**	**2520**	**2520**	**2520**	**2520**	**2520**
stdev	0.00	0.00	0.00	0.00	0.00
**C** *max TI = 2600 ms*	*start PLD = 100 ms*				
rep 0	1600	1520	1520	1520	1521
rep 1	1520	1520	1520	1520	1520
rep 2	1520	1520	1520	1520	1557
**mean**	**1546.67**	**1520.00**	**1520.00**	**1520.00**	**1532.67**
stdev	37.71	0.00	0.00	0.00	17.21

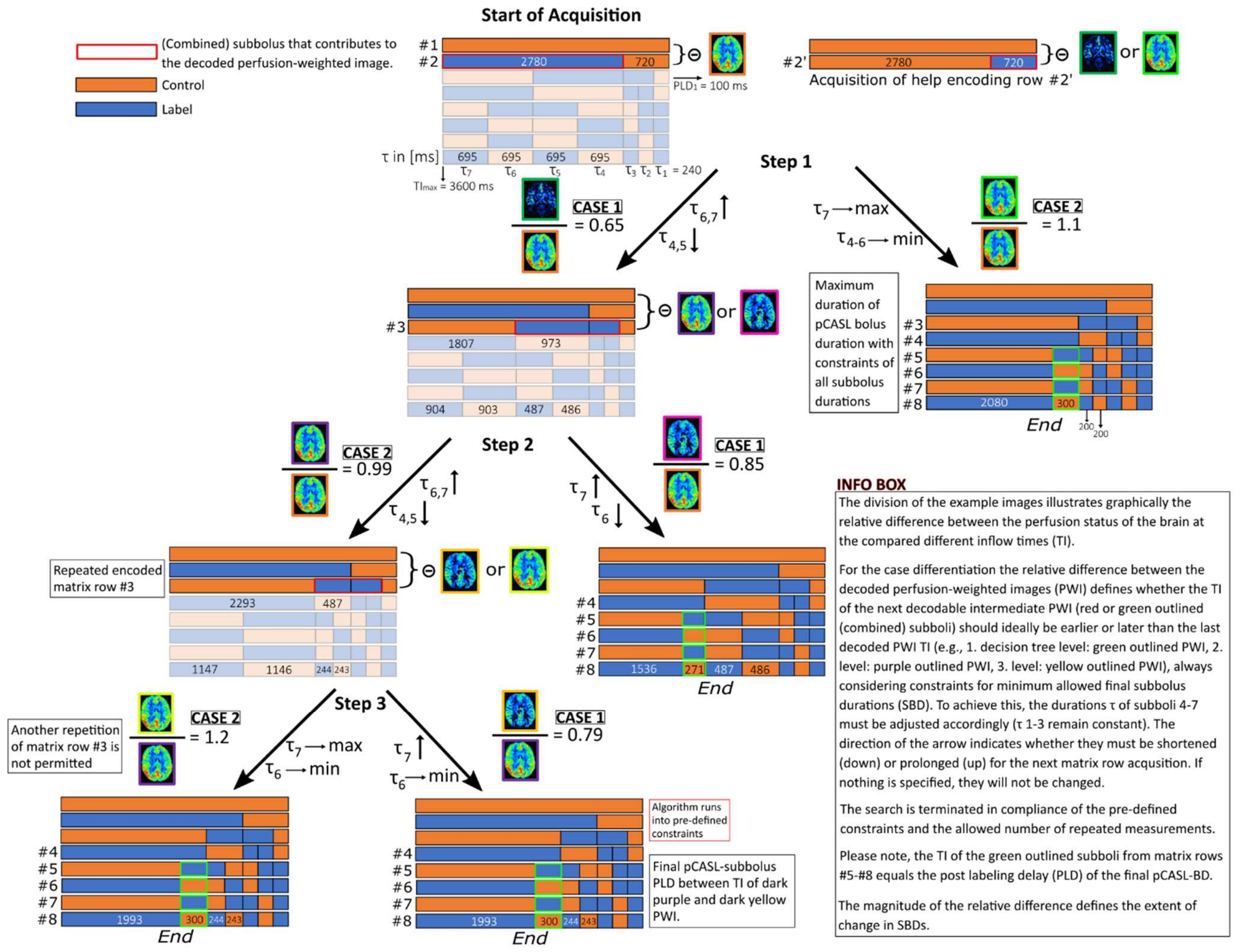

Figure S3: Example values for start and end subbolus durations.
