# Supplementary material for: Subject-specific timing adaption in time-encoded arterial spin labeling imaging
Source: MAGMA. 2023 Sep 28;37(1):53–68. doi: 10.1007/s10334-023-01121-y (PMC10876770; doi:10.1007/s10334-023-01121-y)
Supplement: Supplementary file 2 — Supplementary file2 (PDF 994 KB) [file 10334_2023_1121_MOESM2_ESM.pdf]

Subject-Specific Timing Adaption in Time-Encoded Arterial Spin Labeling Imaging

Dr. rer. nat. Nora-Josefin Breutigam[1], Dr. rer. nat. Daniel Christopher Hoinkiss[1], Dr. rer. nat. Simon Konstandin[1,2], Mareike Alicja Buck[1,3] MSc, Amnah Mahroo[1] MSc, Prof. Dr. rer. nat. Klaus Eickel[1,2,4], Dr. rer. nat. Federico von Samson-Himmelstjerna[1,3], Prof. Dr. rer. nat. Matthias Günther[1,2,3]

[1]Imaging Physics, Fraunhofer Institute for Digital Medicine MEVIS, Bremen, Germany
[2]mediri GmbH, Heidelberg, Germany
[3]Faculty 1 (Physics/Electrical Engineering), University of Bremen, Bremen, Germany
[4]Bremerhaven University of Applied Science, Bremerhaven, Germany

Contact: nora-josefin.Breutigam@mevis.fraunhofer.de

Algorithm adaptive TI time-encoded pCASL

Start

1 Acquire encoded images from **matrix rows #1, #2 and #2'** (= matrix row **#2** with interchanged label and control states) with starting subbolus durations (**SBDs, $\boldsymbol{\tau_i}$** (i = 1,…,7)).

2 Subtract encoded image **#2** from encoded image **#1** = Perfusion-weighted image (**PWI**) **#1**

3 Subtract encoded image **#2'** from encoded image **#1** = **PWI #2**

4 Calculate noise level in **PWI #1 and #2**

5 Count number of voxel above noise level (**NoV**)

6 Compare NoV from **PWI #1** and **#2**

7 Calculate relative difference between NoV:

$$R_{rel\#1} = \frac{NoV_{PWI\#2}}{NoV_{PWI\#1}}$$

If $R_{rel\#1} < 0.9$ case **1** is **true:**

8a Calculate updated **SBDs** for acquisition of encoded image from matrix row **#3**:

$$BD_{pCASL,init} = \sum_{i=4}^{7} \tau_i$$

$$BD_{pCASL,new} = R_{rel\#1} * BD_{pCASL,init}$$

$\tau_{7,new}$ = ceil($BD_{pCASL,new}$ / 2.)
$\tau_{6,new}$ = floor($BD_{pCASL,new}$ / 2.)
$\tau_{5,new}$ = ceil(($BD_{pCASL,init} - BD_{pCASL,new}$) / 2.)
$\tau_{4,new}$ = floor(($BD_{pCASL,init} - BD_{pCASL,new}$) / 2.)
$\tau_{3,new} = \tau_3$
$\tau_{2,new} = \tau_2$
$\tau_{1,new} = \tau_1$

9a Acquire encoded image from matrix row **#3**

10a Subtract encoded image **#3** from encoded image **#1** = **PWI #3**

11a Calculate relative difference between NoV:

$$R_{rel\#2} = \frac{NoV_{PWI\#3}}{NoV_{PWI\#1}}$$

If $R_{rel\#2} < 0.9$ case **1** is **true:**

12a Calculate updated **SBDs** for acquisitions of encoded images from matrix rows **#4** - **#8**:

$BD_{pCASL,old} = \tau_{6,old} + \tau_{7,old}$ ($\tau_{i,old} = \tau_{i,new}$ from **step 8a**)

$BD_{pCASL,new} = R_{rel\#2} * BD_{pCASL,old}$

$\tau_{7,new}$ = ceil($BD_{pCASL,new}$)
$\tau_{6,new}$ = floor ($BD_{pCASL,old} - BD_{pCASL,new}$)
$\tau_{5,new} = \tau_{5,old}$
$\tau_{4,new} = \tau_{4,old}$
$\tau_{3,2,1,new} = \tau_{3,2,1}$

13a Acquire encoded images from matrix rows **#4** - **#8**

End

Else If $R_{rel\#2} \geq 0.9$ case **2** is **true:**

12b Calculate updated **SBDs**: *Acquisition of matrix row **#3** must be repeated.*

$BD_{pCASL,ref} = BD_{pCASL,old}$ ($BD_{pCASL,old} = BD_{pCASL,new}$ from **step 8a**)

$BD_{pCASL,new} = BD_{pCASL,ref} + (0.5 * (BD_{pCASL,init} - BD_{pCASL,ref}))$

$\tau_{7,new}$ = ceil($BD_{pCASL,new}$ / 2.)
$\tau_{6,new}$ = floor($BD_{pCASL,new}$ / 2.)
$\tau_{5,new}$ = ceil(($BD_{pCASL,init} - BD_{pCASL,new}$) / 2.)
$\tau_{4,new}$ = floor($BD_{pCASL,init} - BD_{pCASL,new}$) / 2.)
$\tau_{3,2,1,new} = \tau_{3,2,1}$

13b Repeat acquisition of encoded image from matrix row **#3**

14b Repeat steps **10a & 11a**

If case **1** becomes **true:**

$Diff_{BDpCASL} = BD_{pCASL,old} - BD_{pCASL,ref}$ ($BD_{pCASL,old} = BD_{pCASL,new}$ from **step 12b**)

$BD_{pCASL,new} = BD_{pCASL,old} - (Diff_{BDpCASL} * (1. - R_{rel\#2}))$

$\tau_{7,new}$ = ceil($BD_{pCASL,new}$)
$\tau_{6,new}$ = floor ($BD_{pCASL,old} - BD_{pCASL,new}$)
$\tau_{5,new} = \tau_{5,old}$
$\tau_{4,new} = \tau_{4,old}$
$\tau_{3,2,1,new} = \tau_{3,2,1}$

{$\tau_{i,old} = \tau_{i,new}$ from **step 12b**

If case **2** becomes **true:**

*Allowed number of repetitions of matrix row **#3** is set to 1 in this case (users choice).*

15b Calculate longest possible BD for pCASL bolus at this point:

$\tau_{7,new} = \tau_{7,old} + \tau_{6,old} - 300.$
$\tau_{6,new}$ = 300.
$\tau_{5,new} = \tau_{5,old}$
$\tau_{4,new} = \tau_{4,old}$
$\tau_{3,2,1,new} = \tau_{3,2,1}$

{$\tau_{i,old} = \tau_{i,new}$ from **step 12b**

16b Acquire encoded images from matrix rows **#4** - **#8**

End

Else If $R_{rel\#1} \geq 0.9$ case **2** is **true:**

pCASL BD will be set to the longest possible duration while all remaining subboli are set to the minimum allowed duration, here 300 and 200 ms (users' choice).

8b Calculate updated **SBDs**:

$\tau_{7,new} = (\sum_{i=4}^{7} \tau_i) - 300. - (200. * 2)$
$\tau_{6,new}$ = 300.
$\tau_{5,new}$ = 200.
$\tau_{4,new}$ = 200.
$\tau_{3,2,1,new} = \tau_{3,2,1}$

9b Acquire encoded images from matrix rows **#3** - **#8**

End

Please note: For any adjustment, the minimum allowed (sub)bolus duration must be respected due to T_1 relaxation effects and SNR. This means that if the calculated adaption falls below this, the duration of the pCASL bolus and the other subboli must be adjusted accordingly. In this work, a minimum duration for the pCASL bolus of 1000 ms, for the previous one (τ_6) 300 ms, and for τ_5 and τ_4 200 ms was chosen.

Subbolus durations can be adjusted incrementally during the run time of the measurement using this algorithm. The total duration of the combined bolus (pCASL bolus plus the other subboli) must remain constant. The maximum inflow time can therefore also not change and must be chosen sensibly according to the application. Constant safety checks/testing of this logic during subbolus duration adjustments are recommended.
